# Eave tubes for malaria control in Africa: prototyping and evaluation against *Anopheles gambiae* s.s. and *Anopheles arabiensis* under semi-field conditions in western Kenya

**DOI:** 10.1186/s12936-017-1926-5

**Published:** 2017-07-04

**Authors:** Janneke Snetselaar, Basilio N. Njiru, Beatrice Gachie, Phillip Owigo, Rob Andriessen, Katey Glunt, Anne J. Osinga, James Mutunga, Marit Farenhorst, Bart G. J. Knols

**Affiliations:** 1 In2Care BV, Marijkeweg 22, 6709 PG Wageningen, The Netherlands; 2 London School of Hygiene and Tropical Medicine, Keppel St., Bloomsbury, London, WC1E 7HT UK; 3 International Centre of Insect Physiology and Ecology, Thomas Odhiambo Campus, Mbita Point, 40305 Kenya; 4 Proti-Farm BV, Harderwijkerweg 141B, 3852 AB Ermelo, The Netherlands; 5 Center for Infectious Disease Dynamics and Department of Entomology, The Pennsylvania State University, University Park, USA; 6 Department of Environmental Science, Radboud University Nijmegen, Heyendaalseweg 135, 6525 AJ Nijmegen, The Netherlands

**Keywords:** Eave tubes, Semi-field system, House improvement, Kenya, *Anopheles gambiae*, *Anopheles arabiensis*

## Abstract

**Background:**

Whilst significant progress has been made in the fight against malaria, vector control continues to rely on just two insecticidal methods, i.e., indoor residual spraying and insecticidal bed nets. House improvement shows great potential to complement these methods and may further reduce indoor mosquito biting and disease transmission. Open eaves serve as important mosquito house entry points and provide a suitable location for intercepting host-seeking anophelines. This study describes semi-field experiments in western Kenya with eave tubes, a household protection product that leverages the natural behaviour of host-seeking malaria mosquitoes.

**Methods:**

Semi-field experiments were conducted in two screen-houses. In both of these a typical western Kenyan house, with mud walls and corrugated iron sheet roofing, was built. Eave tubes with bendiocarb- or deltamethrin-treated eave tube inserts were installed in the houses, and the impact on house entry of local strains of *Anopheles gambiae* and *Anopheles arabiensis* was determined. Experiments with open eave tubes (no netting) were conducted as a control and to determine house entry through eave tubes. Insecticidal activity of the inserts treated with insecticide was examined using standard 3-min exposure bioassays.

**Results:**

Experiments with open eave tubes showed that a high percentage of released mosquitoes entered the house through tubes during experimental nights. When tubes were fitted with bendiocarb- or deltamethrin-treated inserts, on average 21% [95% CI 18–25%] and 39% [CI 26–51%] of *An. gambiae* s.s. were recaptured the following morning, respectively. This contrasts with 71% [CI 60–81%] in the treatment with open eaves and 54% [CI 47–61%] in the treatment where inserts were treated with fluorescent dye powder. For *An. arabiensis* recapture was 21% [CI 14–27%] and 22% [CI 18–25%], respectively, compared to 46% [CI 40–52%] and 25% [CI 15–35%] in the treatments with open tubes and fluorescent dye.

**Conclusions:**

Insecticide-treated eave tubes resulted in significant reductions in recapture rates for both malaria vector species, representing the first and promising results with this novel control tool against Kenyan malaria vectors. Further field evaluation of eave tubes under more realistic field conditions, as well as their comparison with existing approaches in terms of cost-effectiveness and community acceptance, is called for.

**Electronic supplementary material:**

The online version of this article (doi:10.1186/s12936-017-1926-5) contains supplementary material, which is available to authorized users.

## Background

The last decade has seen major successes in the global fight against malaria. Long lasting insecticidal nets (LLINs) and indoor residual spraying (IRS), combined with improved diagnosis and effective medication, have saved millions of lives [1]. Nevertheless, despite the impressive progress made in malaria control, the disease remains a substantial global public health problem, with 429,000 deaths (92% of these in Africa) and 212 million cases (90% of these in Africa) in 2015, affecting mostly children and pregnant women [2]. Furthermore, the large-scale use of a limited arsenal of World Health Organization (WHO)-recommended public health insecticides, and impact from agricultural pesticide residues in the environment [3, 4] have resulted in the development of widespread insecticide resistance in mosquitoes [5, 6].

For sustainable vector control the development of new tools and insecticides that successfully target disease-transmitting mosquitoes remains a top priority [7, 8].

House improvements have shown great potential to reduce mosquito biting and decrease the risk for house occupants to contract malaria [9–11]. Since the major African malaria vectors are predominantly endophagic and nocturnal, up to 80–100% of infectious bites occur indoors [12]. Preventing house entry of host-seeking mosquitoes can, therefore, be an effective means to reduce malaria transmission. This was, for instance, demonstrated in a large trial in The Gambia where both fully screened houses or the use of screened ceilings yielded an impressive 50% reduction in anaemia in children under 10 years of age compared to children occupying unscreened housing [13].

Across Africa, traditional house designs are rapidly being replaced with more modern structures. Houses with mud walls and grass-thatched roofs transition to more durable domiciles consisting of concrete or brick walls and corrugated iron sheet roofs [11, 14, 26]. These changes in house design offer new opportunities to interfere with mosquito host-seeking behaviour. An important entry route for *Anopheles* mosquitoes into houses is through the ventilation opening between the wall and the roof, the so-called ‘eave’ [15–18]. Convection heat causes human odour-laden air inside the house to rise. This airstream is funnelled outwards through the eaves at night, which causes attraction of host-seeking mosquitoes. The importance of eaves as the preferred entry point for *Anopheles* mosquitoes, and therefore as a suitable site to intercept these at this stage, has been recognized by WHO since 1997 [19].

House modifications aimed at reducing indoor biting, such as insecticide-treated eave curtains, have shown entomological [20] as well as epidemiological impact [21]. Furthermore, field studies have demonstrated that, compared to houses with open eaves, inhabitants of houses with closed eaves experienced significantly fewer bites indoors and had a lower risk of malaria infection [16, 22–24]. A trial with permethrin-treated eave curtains (in which the entire eave gap was closed with netting material and doors and windows were screened) in Burkina Faso showed a 15% reduction in child mortality [25]. There is good evidence and justification, therefore, to focus on eaves as a point to interrupt the life cycle of anophelines and in doing so prevent malaria transmission.

In the present study the eave tube concept that targets malaria mosquitoes at eave level was evaluated. The basic principle of the eave tube concept is to limit mosquito access to the house by screening or blocking openings where feasible and adding tubes fitted with removable gauze inserts [26, 27]. Installation of eave tubes starts by rendering houses mosquito proof through sealing of the eaves and screening the windows and subsequently introducing openings in the wall at eave level, which leverage the natural route of host-seeking mosquitoes to enter houses at night through open eaves. When mosquitoes enter an eave tube they encounter a netting barrier, consisting of an insert fitted with insecticide-treated electrostatic netting. Previous research has shown that this electrostatic netting can bind a variety of insecticides and provides enhanced bioavailability of these insecticides [28, 29]. A major benefit of eave tubes is that they work passively, not requiring active engagement from house occupants, as is the case with LLINs, which require daily involvement when in use. Installed beyond the reach of house occupants they enable safe use of insecticides, including the use of actives that are not (yet) recommended for IRS or LLINs, which opens options for using alternative insecticides and biopesticides such as the entomopathogenic fungus *Beauveria bassiana,* which may be effective against pyrethroid-resistant populations. Here, it is shown how electrostatic netting, when used inside eave tubes provides a novel delivery tool for insecticidal agents to target malaria vectors.

This study evaluated the use of eave tubes in an experimental semi-field setting in Kenya, where prototype tubes and inserts were fitted into a replicate of a local mud-walled house with corrugated iron roofing sheets. The impact of insecticide-treated eave tube inserts was evaluated through assessment of exposure rates and mortality impact using two species of Kenyan anophelines. House entry of mosquitoes through open eave tubes (without netting) was recorded to assess responses of mosquitoes towards tubes as house entry points.

## Methods

### Experimental set-up

**Fig. 1:**
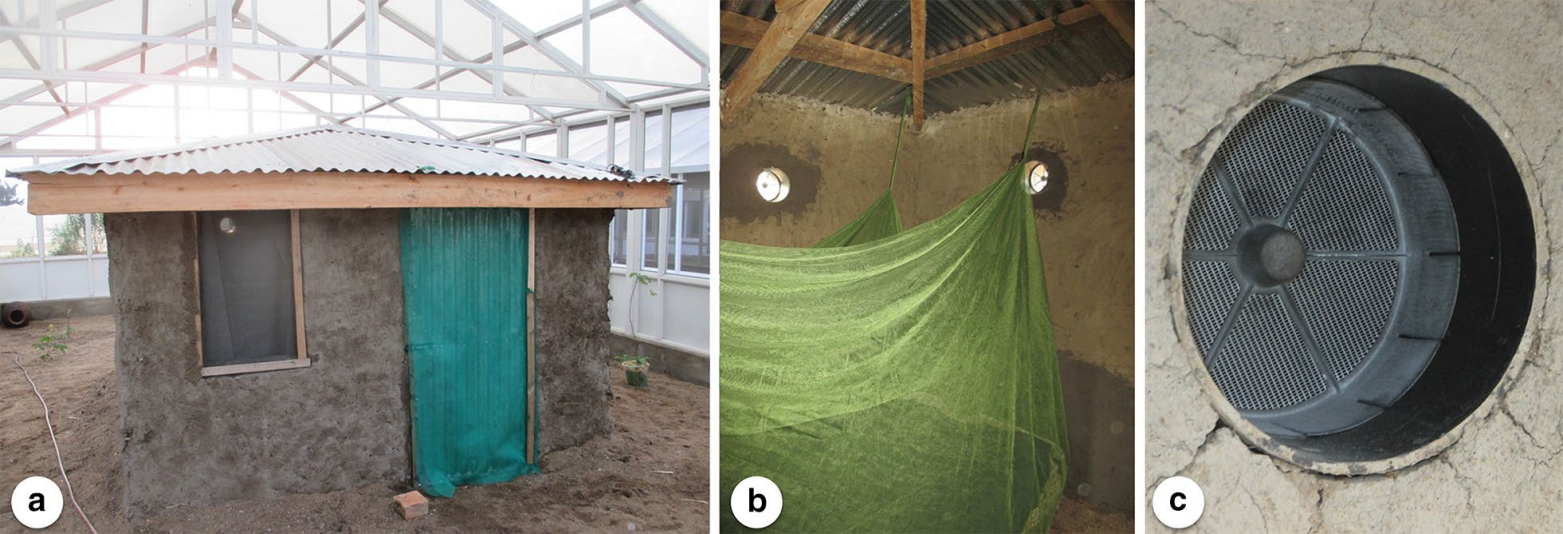
**a** Experimental house (3 × 3 m) inside screen-house with mud wall, corrugated iron roofing, screened window and door. **b** Inside the house with untreated bed net, sealed eaves and eave tubes. **c** Eave tube with treated insert as seen from the outside

The study was carried out at the International Centre of Insect Physiology and Ecology (ICIPE) located at Mbita Point, western Kenya (0°26′06.19″S, 34°12′53.13″E). Two semi-field screen-houses, both 7.1 × 11 m in size, were used in parallel. Screen-houses, i.e. large outdoor enclosures covered with netting, were designed to simulate a natural ecosystem for anopheline mosquitoes, as previously described by Knols et al. [30], had sandy soil, little vegetation, and mosquito resting places that consisted of moist clay pots [31]. In each of the screen-houses an experimental house of 3 × 3 m was constructed and fitted with a single bed in which a volunteer slept under an untreated bed net during experimental nights (Fig. 1a, b). The houses resembled a local rural design with walls made out of mud and a roof made out of corrugated iron sheets as is commonly seen in western Kenya. A mixture of wood, ash and clay was used for plastering and smoothing the wall surfaces. Openings and cracks in the walls were sealed with clay and the window was fitted with untreated mosquito-proof netting and a cotton curtain. In each hut the eaves were sealed and a total of six black 6-in. PVC pipes were installed at 1-min intervals under the roof at eave height in each house (Fig. 1c).

### Eave tube prototypes

**Fig. 2:**
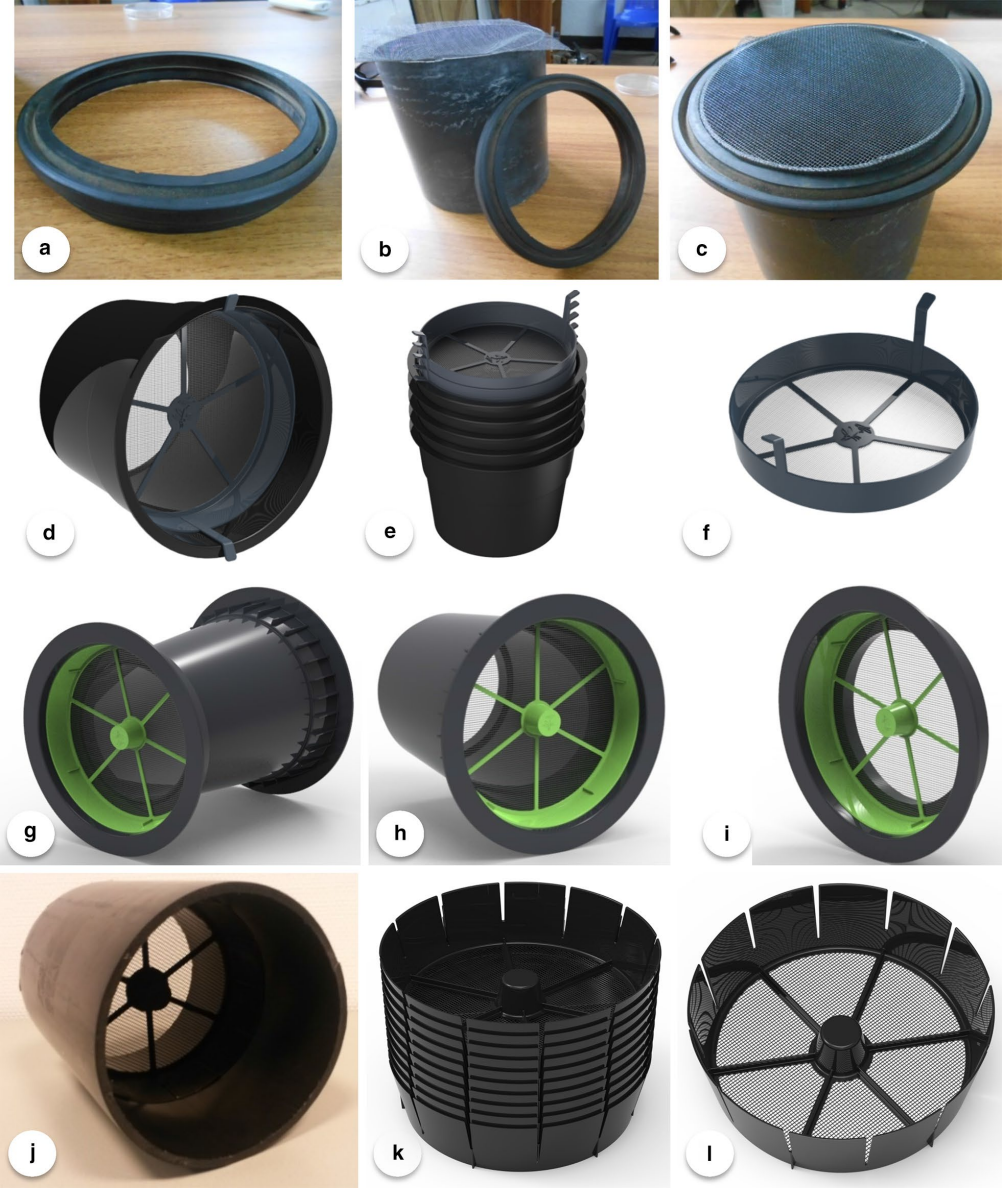
Eave tube prototypes. Originally, electrostatic netting was fitted over the PVC pipe using a rubber or PVC ring (**a**–**c**). Subsequently a second generation of tubes with special inserts (**d**–**f** and **g**–**i**) was developed. Unfortunately these inserts were too close to the outside of the house, which resulted in the development of an eave tube insert that can slide inside the PVC pipe (**j**), can easily be stacked (**k**), and is slightly conical to fit in different diameter tubes (**l**). 250 of these eave tube inserts (**l**) can be packed in a box of 60 × 40 × 40 cm

As the concept of eave tubes for malaria vector control progressed, alternative designs with varying features and characteristics were developed and tested (Fig. 2a–l). Following experimentation on tube size in Tanzania [27, and Fig. 2b therein], eave tube inserts were designed to fit in 6-in. PVC pipes, which are widely available across Africa. The inserts used in this study were 45 mm high and conically shaped, with a diameter of 144 mm at the bottom end and 156 mm at the top end (Fig. 2l). Because the polypropylene is flexible, the inserts fit in PVC pipes of variable thickness, without allowing space for mosquitoes to pass. The spokes in the insert provide additional support to enable handling, stacking (Fig. 2k) and placement of the insert in tubes. In this study, black 20 cm long, PVC pipes were used. The inserts were placed halfway into the tubes (Fig. 2j) to prevent direct exposure to sunlight. A video that shows how an insert is being installed inside a PVC pipe is shown in Additional file 1.

### Mosquitoes

Experiments were conducted with *Anopheles gambiae* s.s. and *Anopheles arabiensis* mosquitoes that were colonized from specimens that originated from the Mbita area. Larvae were reared in filtered water from Lake Victoria and fed twice a day on locally available cat food. Pupae were collected daily and placed into 30 × 30 × 30 cm netting cages until emergence. Adults were offered ad libitum access to a 6% glucose solution and were fed on blood from adult human volunteers that were examined twice a week for the presence of malaria parasites. Mosquitoes were reared under ambient climatic conditions in a screen-house similar to the ones in which experiments were conducted. Prior to the experiments, host-seeking female mosquitoes were selected from adult holding cages and placed in cylindrical release cups.

### Eave tube treatment

Eave tube inserts were fitted with netting with an electrostatic coating that binds insecticidal particles [28]. Because the electrostatic coating enables adherence of particles without a carrier or impregnation process, a large variety of active compounds can be used. Here, orange fluorescent dust (BVDA International BV, Haarlem, The Netherlands) and two insecticides widely used in malaria vector control, i.e., bendiocarb (Ficam D, 1.25% a.i., Bayer, Leverkusen, Germany) and deltamethrin (Spritex 0.25% a.i., Denka International BV, Barneveld, The Netherlands) were used. Fluorescent or insecticide powder formulations were applied on electrostatic netting by placing the inserts in a closed bucket with an excess amount of formulation and shaking it for 15–30 s until the inserts were saturated.

### Exposure bioassays

Exposure bioassays were conducted before the experiments according to WHO protocol [32] and were similar to the MCD bottle assay described by Sternberg et al. [33]. Insecticidal activity of the inserts saturated with insecticide was checked using 3-min exposure assays with 50 host-seeking female mosquitoes, exposed in cohorts of five to ten individuals. Knockdown (1 h post exposure) and mortality rates (24 h post exposure) were recorded. Bioassays were conducted with fluorescent dust (control treatment) or bendiocarb/deltamethrin dust applied onto the electrostatic netting. Baseline exposure doses were measured by determining the presence and amount of fluorescent particles on the exposed mosquito bodies, by killing the females after exposure and checking them using a UV light microscope (Dino Lite Premier). A second series of exposures was performed at the end of the semi-field trials with the inserts that had been used for three to five consecutive weeks in order to ascertain that the residual activity of the insecticides remained unchanged throughout the experimental period.

Results from the semi-field bioassays indicated that temperature could have an impact on insecticidal impact on mosquitoes. To examine the influence of temperature on survival following bendiocarb or deltamethrin exposure, non-blood fed, 5-day-old *An. gambiae* females were exposed to these insecticides at 18 or 27 °C using the MCD bottle bioassay at Penn State University. Mosquitoes were from a single cohort that had been reared to adults at a constant 27 °C. Females were moved to environmental chambers set to 18 or 27 °C and 85% RH 15–30 min prior to exposure. For each of five replicates, five mosquitoes were aspirated into an MCD bottle fitted with either untreated electrostatic netting, or netting treated with the same bendiocarb or deltamethrin formulations as used in Kenya. A glass bottle filled with hot water served as an attractant source and exposures lasted 1 min. Once removed from the MCD bottle, mosquitoes were kept at treatment temperature for 24 h. Mortality was assessed after 24 h; mosquitoes that were moribund or unable to fly were scored as dead.

### Screen-house experiments

The two mosquito species were tested in succession, the first 3 weeks (6 replicates) focused on *An. gambiae* s.s. and the following 5 weeks (13 replicates) on *An. arabiensis.* Larger variation in control treatment recaptures for *An. arabiensis* necessitated more replicates. For each species, fresh tube inserts with actives were prepared and installed at the start of the experimental series. Before each experimental night, eave tube inserts were placed inside the tubes in the experimental houses, after which 200 host-seeking female mosquitoes were released outside the houses at 19.00 h local time. A sleeper was present inside the house under an untreated bed net to serve as bait for the host-seeking mosquitoes. Two rounds of collections were done the following morning at 07.00 h and at 12:00 h according to normal practice at the research site when conducting screenhouse experiments. One technician per screenhouse collected mosquitoes for 1 h both inside and outside the houses using a backpack aspirator and recorded the numbers recaptured. After each experiment the inserts were removed from the houses and stored at ambient temperature in the laboratory. These inserts were (re-) used six times over a period of approximately 3 weeks.

Two different methods were used to determine house entry through eave tubes: (a) open eave tubes (PVC only without installing the eave tube inserts); and, (b) eave tube inserts treated with fluorescent dye that served as a proxy for contact with insecticidal netting during the experimental night [see 29 and Fig. 5 therein]. During experiments with open tubes, mosquitoes could freely enter the house and the number that entered the house was determined through indoor collections using both a standard CDC miniature light trap (John W Hock Co., USA) positioned next to the bed net [34] and a backpack aspirator. Attraction of eave tubes was assessed by treating the netting with fluorescent powder, a non-lethal marker [35] to measure the proportion of females released that contacted the netting installed in the eave tube. Mosquitoes bearing fluorescent dye were scored using a UV light microscope (Dino Lite Premier, USA).

The experiments reported here differ from those reported from semi-field studies in Tanzania [27] in three ways. First, unlike the experimental (wooden) huts that were used in Tanzania, the work in Kenya was conducted in copies of real housing structures found in western Kenya that consisted of mud walls and corrugated iron sheet roofing. Second, eave tubes in Tanzania were covered with (treated) netting, whereas the Kenya studies used inserts that can be mass-produced (Fig. 2l). Finally, the Kenya study differed in that it not only incorporated another (Kenyan) strain of *An. arabiensis* but also, for the first time, a local strain of *An. gambiae* s.s.

### Climate data

To measure exposure conditions, climate data from data loggers (VOLTCRAFT DL-121TH, Conrad Electronic Benelux BV, Oldenzaal, The Netherlands) placed inside and outside the experimental houses was recorded. Both data loggers were suspended 0.5 m below the edge of the ceiling and temperature and humidity were recorded during experimental hours at 30-min intervals.

### Data analysis

Raw data was collected on daily record sheets and entered into an Excel spreadsheet the following day. Data was available to all involved in the research via an online platform. Impact of the insecticides was calculated by comparing the numbers of retrieved mosquitoes for the different treatments and the controls. Differences in recapture numbers served as an indicator for the potential vector control impact of eave tubes achieved within a single night [27].

Data were analysed using SPSS 21.0 software. Normality of the data was investigated using the Shapiro–Wilk test and homogeneity of variances was tested with Levene’s Test (untransformed data). Treatments were compared using the Mann–Whitney U test with Bonferroni correction for multiple comparisons.

R software (version 3.2.1; the R Foundation for statistical computing, Austria) was used to analyse the effect of temperature on insecticide-induced mortality. Fixed effects were insecticide exposure (control or bendiocarb/deltamethrin), temperature (18 or 27 °C) and the interaction between these parameters.

## Results

### Exposure bioassays

At the onset of the experimental series, exposure bioassays with both bendiocarb and deltamethrin yielded 100% knockdown (1 h) and 100% mortality (24 h) for both mosquito species (four replicates in total; Table 1). Fluorescent dust was used as a control treatment and exposure to inserts treated with fluorescent dye resulted in mortality lower than 20% in all bioassays (Table 1). In all replicates 100% of mosquitoes were contaminated with fluorescent dust, confirming that the bioassay method was effective for exposing mosquitoes in the screenhouse experiments.

**Table 1:** Knockdown (1 h) and mortality (24 h post exposure) of *Anopheles gambiae* s.l. mosquitoes exposed to insecticide-treated eave tube inserts for 3 min before or after (3–5 weeks) the screen-house experiments (4 replicates per treatment)

		Before screen-house tests		After screen-house tests	
Species	Eave tube insert treatment	Knockdown average % [±95% CI]	Mortality average % [±95% CI]	Knockdown average % [±95% CI]	Mortality average % [±95% CI]
*An. gambiae*	Fluorescent dust	6.5 [2.4–10.6]	17.8 [7.3–28.4 ± 5.4]	0	4.1 [0.9 to 7.2]
	Deltamethrin (0.25%)	100	100	100	100
	Bendiocarb (1.25%)	100	100	98.9 [96.9–101.1]	98.9 [96.9 to 101.1]
*An. arabiensis*	Fluorescent dust	0	1.0 [−0.1 to 2.2]	0	1.8 [−1.7 to 5.3]
	Deltamethrin (0.25%)	100	100	98.5 [96.9–100.4]	96.6 [93.2 to 99.9]
	Bendiocarb (1.25%)	100	100	33.4 [9.7–57.2]	38.8 [11.2 to 66.3]

After extensive handling of the inserts during the screen-house experiments, deltamethrin and bendiocarb-treated inserts still resulted in 99–100% *An. gambiae* s.s. mortality in the exposure assays. For *An. arabiensis* the efficacy of bendiocarb-treated inserts reduced sharply after the screen-house experiments, resulting in only 33% knockdown and 39% mortality.

**Fig. 3:**
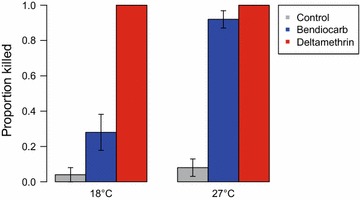
Mortality of *An. gambiae* s.s. 24 h after a 1-min exposure to control or insecticide-treated netting at 18 or 27 °C. While deltamethrin killed all mosquitoes regardless of temperature, bendiocarb was significantly less lethal at 18 than at 27 °C (p < 0.001)

Interestingly, there was a clear effect of temperature on the efficacy of bendiocarb on *An. gambiae* females exposed using the MCD bottle. While deltamethrin killed 100% of exposed mosquitoes regardless of temperature, bendiocarb gave on average a 60% lower mortality (Χ^2^ = 20.8, df_1,19_, p < 0.001) at 18 than at 27 °C (Fig. 3).

### Response to eave tubes

**Fig. 4:**
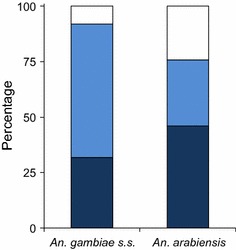
House entry by mosquitoes through open eave tubes. For *An. gambiae* s.s., 92% of the released mosquitoes were retrieved indoors (*light blue* backpack aspirator, *dark blue* CDC light trap, the rest outdoors (*white* backpack aspirator). For *An. arabiensis*, indoor captures totalled 76%

Experiments with open eave tubes (i.e., PVC pipes only without eave tube inserts to prevent house entry) were conducted to measure responses of both species towards eave tubes as house entry points. For the series with *An. gambiae* s.s, an average of 71% [95% CI 60–81%] of the mosquitoes released each night were recaptured. Of these, 92% were caught indoors, out of which 31% was recaptured with the CDC light trap and 61% with the backpack aspirator. For *An. arabiensis*, overall recapture was lower at 46% [CI 40–52%], of which 76% was recaptured indoors (Fig. 4).

CDC light trap catches were smaller than expected and comprised less than half of the total mosquito numbers retrieved. Use of this recapture technique alone would have caused an underestimation of the number of mosquitoes that entered the house, which is why backpack aspiration was included.

The second method to determine mosquito responses to eave tubes was based on colour marking of mosquitoes with fluorescent dye when making contact with tube netting [29]. Following an experimental night, mosquitoes were recaptured, killed and examined for the presence of fluorescent dust using a UV light microscope. On average 41% [CI 32–51%] of the recaptured *An. gambiae* s.s. mosquitoes had fluorescent dust on their bodies, indicating that they had contact (at least once) with the fluorescent-dust-treated inserts. For *An. arabiensis* this was 30% [CI 21–40%].

### Insecticide treatments

Results showed a significant impact on mosquito mortality when eave tube inserts were treated with insecticide. Out of 200 *An. gambiae* s.s. mosquitoes released each night, significantly fewer were recaptured the following morning when deltamethrin-treated inserts were used, compared to the control treatments (with open eave tubes; p = 0.002, Mann–Whitney U test, Bonferroni corrected; Table 2) and the control treatment with fluorescent dye (p = 0.002). No significant difference was found for inserts treated with bendiocarb compared to the fluorescent dust treatment. After an experimental night with deltamethrin-treated inserts, on average 21% [CI 14–29%] of the released mosquitoes was recaptured compared to 71% [CI 60–81%] in the control treatment with open eave tubes; for bendiocarb-treated inserts this was 39% [CI 26–51%].

**Table 2:** Percentage mosquitoes recaptured (±95% CI) in screenhouse experiments with *An. gambiae* and *An. arabiensis*

Species	Treatment	%	[95% CI]	# released	# recaptured	p values	
*An. gambiae*	Open	70.8	[60.3–81.4]	1200	850	–	0.041
	FD	54.1	[47.3–60.8]	1200	649	0.041	–
	BC	38.8	[26.2–51.3]	1200	465	0.009	0.065
	DM	21.3	[14.0–28.7]	1200	256	0.002	0.002
*An. arabiensis*	Open	45.8	[39.7–51.8]	2600	1190	–	0.020
	FD	25.2	[14.9–35.4]	2600	654	0.020	–
	BC	20.6	[14.4–26.9]	2600	536	<0.001	0.762
	DM	22.0	[18.1–25.0]	2600	572	<0.001	0.614

Treatments included two controls; open eave tubes (Open) or inserts with fluorescent dye (FD). Controls were compared to treatments with bendiocarb (BC) or deltamethrin (DM)-treated inserts using Mann–Whitney U tests

Results for *An. arabiensis* (Table 2) were similar, although the impact of the insecticide treatments was smaller. After an experimental night with deltamethrin-treated inserts, on average 22% [CI 18–25%] of the released mosquitoes was recaptured compared to 46% [CI 40–52%] in the control treatment with open eave tubes; for bendiocarb-treated inserts this was 25% [CI 15–35%]. Insecticide treatments were significantly different from the control with open eave tubes (p < 0.001) but not different from the control with fluorescent powder. Raw data on the recapture of *An. gambiae *and *An. arabiensis* during experimental nights with either control or insecticide treatments is provided in Additional file 2.

### Climate data

**Fig. 5:**
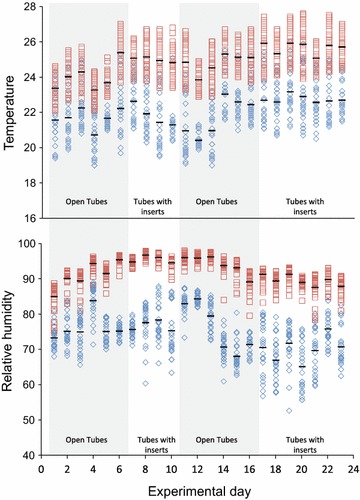
Temperature and relative humidity inside (*squares*) and outside (*diamonds*) the experimental house during the experimental period. Measurements of temperature and humidity were taken at 30 min intervals. Environmental data was collected between 19:00 and 7:00 h. Horizontal stripes represent the average per experimental night

Both temperature and humidity (Fig. 5) were, on average, higher inside the experimental houses compared to outside. Average temperature inside was 2.8 °C higher, for relative humidity there was a 17.7% difference. A small but significant, on average 0.8 °C, increase in temperature inside the house was observed after placing eave tube inserts in the pipes, compared to open pipes (p < 0.001, Mann–Whitney U test); no significant difference was found for relative humidity.

## Discussion

Here, the first evaluation of eave tube technology under semi-field conditions against Kenyan strains of insecticide-susceptible *An. gambiae* s.s. and *An. arabiensis* (Mbita strains) is presented. It was shown that houses commonly found in western Kenya, constructed out of mud walls with corrugated iron sheet roofing, are highly suitable for this approach. Both species responded strongly to host odours emanating from the tubes as was noted by the high number of mosquitoes recaptured indoors when the tubes were left open, as well as the considerable number that contacted the inserts treated with fluorescent dust. Interestingly, the response of *An. gambiae* s.s. to eave tubes was considerably higher than that of *An. arabiensis*, which may be explained by the generally more endophilic and endophagic nature of the former species [36]. Noteworthy is also the fact that the response of both strains decreased when the tubes were closed with inserts. It is likely that the reduced airflow through the inserts may be a cause for this reduction. Since the netting in the inserts used was quite dense (air permeability of 1600 L/sq m/sec at 20 Pa; ISO 9237:1995) and airflow attenuation can already be considerable with the more open mesh sizes used in commercial LLINs [37], an improvement of tube attraction with more open mesh sizes is foreseen. This reduced airflow also affected indoor climate, which made the house slightly warmer (ca. 0.8 °C) during the night, as well as slightly more humid (ca. 1.3% RH). The more pronounced difference in humidity between the indoor and outdoor environment may be explained by the fact that the houses had only recently been constructed in the screen-houses so that the walls were giving off substantial amounts of water vapour present in the mud during the building stage. Alternatively, or in addition, the presence of a human host in this relatively small house may have caused an increase in indoor humidity through exhaled air. Overall, the differences in indoor climate caused by the installation of eave tubes was minimal and similar to observations from Tanzania (data not published).

Two insecticides commonly used in vector control, deltamethrin and bendiocarb, were applied in powder formulation on the electrostatic netting of the eave tube inserts and tested in the PVC pipes. Both treatments resulted in significant reductions in the number of retrieved mosquitoes compared to the control treatment with open eave tubes; albeit less so when compared with the dye treatments. These findings are in line with the results obtained in screen-house studies in Tanzania [27]. Given the large proportion of mosquitoes around a house that can be targeted with eave tubes, it is foreseen that with substantial coverage and in combination with LLINs they can have a big impact on mosquito populations and hence on malaria transmission [38].

The impact of bendiocarb on mosquito mortality when exposed at different temperatures gives rise to concern regarding the validity of insecticide-resistance assays being performed under a range of insectary and field temperatures. It is also surprising that this phenomenon was not apparent for the pyrethroid insecticide but more so for the carbamate. In essence, this calls for the evaluation of insecticidal impact under different temperature regimes and standardization of climate conditions under which exposure bioassays occur.

Mosquitoes were effectively blocked from the house after the windows were screened with untreated netting and treated eave tube inserts were fitted in the PVC pipes. The doors of the experimental houses were always kept closed during the experiments, and it can be argued that this may not always be so under realistic field conditions. However, even though doors that are kept open during dusk and the night will allow entry by mosquitoes, these belong mostly to the nuisance biting culicines and few will transmit malaria [15, 16, 31]. During the day the house heats up, creating upward convection currents at night that carry body odours upward towards the eaves. Thus, even when the doors are open(ed) and windows are not or only partially screened, the majority of malaria mosquitoes will still enter through the eaves and use it as their primary entry point into the house. This was confirmed by field data from the Kilombero valley in Tanzania, where >90% reduction in indoor mosquito numbers was achieved after houses were rendered mosquito proof and eave tubes were installed [27].

The total surface area of treated eave tube insert netting is very small and only a fraction of the surface sprayed during IRS or even the total surface of an LLIN, which means that much less insecticide will be needed. This will have an impact on cost, and it was estimated that eave tubes are roughly half the cost of IRS (Knols et al., in prep.). Moreover, the >95% reduction in the amount of insecticide needed per house will have major implications on operational costs, particularly when cheaper pyrethroids are being replaced by the more expensive carbamates (bendiocarb) or organophosphates (e.g., pirimiphos-methyl). Ultimately, this can greatly reduce the impact of insecticide costs on control campaigns, which for IRS at present is estimated at 24% [39].

An additional benefit of eave tube technology is that it provides protection for everyone residing indoors. This protection will be longer and more pronounced than bed nets that only protect sleepers during the hours of active use. Moreover, bed net occupants will have daily exposure to pyrethroids when occupying the net, which is overcome when eave tubes are used that are beyond the reach of house occupants. Eave tubes therefore provide a safe means for using insecticides in the vicinity of humans with minimal exposure risk, which opens up possibilities to use actives that would not normally be recommended for wall spraying or bed net impregnation. In turn, this will open opportunities to manage insecticide resistance through the development of inserts that hold more than one active, or use of inserts in houses with different treatments, so that the vector population is constantly exposed to a variety of insecticides [40]. The eave tube design that was used in this study was recently taken into mass production. Further optimizations and development of treatment, washing and re-treatment production systems provide opportunities for development of a product that can be cost-competitive, user-friendly and has impact on malaria vectors comparable to or better than existing vector control tools, notably IRS. This may include the development and production of eave tube inserts out of biodegradable plastics or even re-cycled LLINs.

Besides an effect on malaria vectors, eave tube technology will also provide options to reduce indoor densities of nuisance mosquitoes and exposure to other disease vectors. It was previously found that screening house-entry points also has an effect on indoor densities of vectors of neglected tropical diseases (NTDs), such as lymphatic filariasis, dengue, leishmaniasis [41, 42] and even trachoma and fly-transmitted diarrhoeal disease. This means that eave tubes could contribute to an integrated vector control programme aimed at reducing multiple vector-transmitted diseases [43]. Overall, the technology responds to a dire need for additional measures beyond LLINs and IRS to further the goals of malaria eradication and control of NTDs.

Deployment of LLINs has previously been shown to induce community-wide effects when coverage is high, thereby reducing malaria transmission even in houses that remain unprotected [18, 25]. This advocates for the implementation of mosquito-killing agents, rather than only improving houses with untreated physical barriers. As with LLINs and IRS, eave tubes (in combination with window/door screening) are expected to induce community-wide effects, whereby houses that are unsuitable for eave tube installation still benefit from the technology. Determining what level of coverage is necessary to reduce transmission overall will help inform implementation strategies [38, 44].

## Conclusions

The proposed introduction of eave tube technology (i.e. closing of eaves, installation of eave tubes, and rendering houses mosquito-proof through window/door screening) for malaria control in Africa [26] has so far been supported with encouraging data from semi-field studies in Tanzania [27], which are corroborated with the outcomes of the present semi-field study with Kenyan strains of *An. gambiae* s.s. and *An. arabiensis*. Given the large numbers of females of both strains that were attracted to the eave tubes when responding to host odours emanating from the house, it is likely that similar responses may occur under natural field conditions and hence large numbers of female mosquitoes can be killed by insecticide. There is a need to maintain caution, as this work represents semi-field studies with strains of mosquitoes that have been kept under artificial conditions for several years. Confirmation of these findings in open field studies is therefore required, as well as provision of epidemiological, social and economic evidence that the approach can impact on malaria under real-life conditions [44]. Direct observations on the behaviour of wild mosquitoes inside eave tubes in village houses will be a follow-up article in this series.

## Additional files

**Additional file 1.** Video showing the installation of an eave tube insert in a PVC pipe.

**Additional file 2.** Raw data of the reported experiments.

## Abbreviations

RH: relative humidity
IRS: indoor residual spraying
LLIN: long-lasting insecticide-treated bed net
MCD: mosquito contamination device
MDG: Millennium Development Goal
NTD: neglected tropical diseases
WHO: World Health Organization
