# Supplementary material for: Eave tubes for malaria control in Africa: prototyping and evaluation against Anopheles gambiae s.s. and Anopheles arabiensis under semi-field conditions in western Kenya
Source: Malar J. 2017 Jul 4;16:276. doi: 10.1186/s12936-017-1926-5 (PMC5545004; doi:10.1186/s12936-017-1926-5)
Supplement: Supplementary file 2 — Additional file 2. Raw data of the reported experiments. [file 12936_2017_1926_MOESM2_ESM.docx]

| **Trial day** | **Date** | **Species** | **#Rel** | **#Recap** | **Treatment** | **Time rel** | **Time recap** | **Screenh** | **Sleeper** | **Data logger** |
| --- | --- | --- | --- | --- | --- | --- | --- | --- | --- | --- |
| 1 | 16-Jul | *An. gambiae s.s.* | 200 | 102 | FL dye | 19:00 | 7:00 | 8B | K | - |
| 2 | 17-Jul | *An. gambiae s.s.* | 200 | 127 | FL dye | 19:00 | 7:15 | 8A | M | - |
| 3 | 18-Jul | *An. gambiae s.s.* | 200 | 109 | FL dye | 19:00 | 7:10 | 8B | K | - |
| 4 | 20-Jul | *An. gambiae s.s.* | 200 | 93 | FL dye | 19:00 | 7:00 | 8A | M | DL |
| 5 | 20-Jul | *An. gambiae s.s.* | 200 | 106 | open tubes | 19:00 | 7:00 | 8B | K | - |
| 6 | 21-Jul | *An. gambiae s.s.* | 200 | 156 | open tubes | 19:10 | 7:10 | 8A | M | - |
| 7 | 21-Jul | *An. gambiae s.s.* | 200 | 89 | FL dye | 19:10 | 7:10 | 8B | K | DL |
| 8 | 22-Jul | *An. gambiae s.s.* | 200 | 30 | DM | 19:00 | 7:00 | 8A | M | DL |
| 9 | 22-Jul | *An. gambiae s.s.* | 200 | 45 | BC | 19:00 | 7:00 | 8B | K | - |
| 10 | 23-Jul | *An. gambiae s.s.* | 200 | 82 | BC | 19:00 | 7:00 | 8A | M | - |
| 11 | 23-Jul | *An. gambiae s.s.* | 200 | 21 | DM | 19:00 | 7:00 | 8B | K | DL |
| 12 | 24-Jul | *An. gambiae s.s.* | 200 | 54 | DM | 19:00 | 7:00 | 8A | M | DL |
| 13 | 27-Jul | *An. gambiae s.s.* | 200 | 129 | FL dye | 19:10 | 7:10 | 8A | M | - |
| 14 | 27-Jul | *An. gambiae s.s.* | 200 | 161 | open tubes | 19:10 | 7:10 | 8B | K | DL |
| 15 | 28-Jul | *An. gambiae s.s.* | 200 | 58 | BC | 19:05 | 7:10 | 8A | M | DL |
| 16 | 28-Jul | *An. gambiae s.s.* | 200 | 35 | DM | 19:05 | 7:10 | 8B | K | - |
| 17 | 29-Jul | *An. gambiae s.s.* | 200 | 72 | DM | 19:05 | 7:05 | 8A | M | - |
| 18 | 29-Jul | *An. gambiae s.s.* | 200 | 63 | BC | 19:05 | 7:05 | 8B | K | DL |
| 19 | 30-Jul | *An. gambiae s.s.* | 200 | 83 | BC | 19:00 | 7:10 | 8A | M | DL |
| 20 | 30-Jul | *An. gambiae s.s.* | 200 | 44 | DM | 19:00 | 7:10 | 8B | K | - |
| 21 | 31-Jul | *An. gambiae s.s.* | 200 | 164 | open tubes | 19:05 | 7:05 | 8A | M | - |
| 22 | 31-Jul | *An. gambiae s.s.* | 200 | 134 | BC | 19:05 | 7:05 | 8B | K | DL |
| 23 | 04-Aug | *An. arabiensis* | 200 | 52 | open tubes | 19:15 | 7:15 | 8A | M | - |
| 24 | 04-Aug | *An. arabiensis* | 200 | 141 | FL dye | 19:15 | 7:15 | 8B | K | DL |
| 25 | 05-Aug | *An. arabiensis* | 200 | 51 | FL dye | 19:20 | 7:05 | 8A | M | DL |
| 26 | 05-Aug | *An. arabiensis* | 200 | 134 | open tubes | 19:20 | 7:05 | 8B | K | - |
| 27 | 06-Aug | *An. arabiensis* | 200 | 94 | open tubes | 19:10 | 7:05 | 8A | M | - |
| 28 | 06-Aug | *An. arabiensis* | 200 | 114 | FL dye | 19:10 | 7:05 | 8B | K | DL |
| 29 | 10-Aug | *An. arabiensis* | 200 | 27 | FL dye | 19:10 | 7:00 | 8A | M | - |
| 30 | 10-Aug | *An. arabiensis* | 200 | 82 | open tubes | 19:10 | 7:00 | 8B | K | DL |
| 31 | 12-Aug | *An. arabiensis* | 200 | 109 | open tubes | 19:10 | 7:10 | 8A | M | - |
| 32 | 12-Aug | *An. arabiensis* | 200 | 24 | FL dye | 19:10 | 7:10 | 8B | K | DL |
| 33 | 13-Aug | *An. arabiensis* | 200 | 60 | FL dye | 19:10 | 7:10 | 8A | M | DL |
| 34 | 13-Aug | *An. arabiensis* | 200 | 93 | open tubes | 19:10 | 7:10 | 8B | K | - |
| 35 | 14-Aug | *An. arabiensis* | 200 | 25 | BC | 19:10 | 7:10 | 8A | M | - |
| 36 | 14-Aug | *An. arabiensis* | 200 | 33 | DM | 19:10 | 7:10 | 8B | K | DL |
| 37 | 15-Aug | *An. arabiensis* | 200 | 19 | DM | 19:10 | 7:10 | 8A | M | DL |
| 38 | 15-Aug | *An. arabiensis* | 200 | 26 | BC | 19:10 | 7:10 | 8B | K | - |
| 39 | 16-Aug | *An. gambiae s.s.* | 200 | 110 | open tubes | 19:15 | 7:10 | 8A | M | - |
| 40 | 16-Aug | *An. gambiae s.s.* | 200 | 153 | open tubes | 19:10 | 7:10 | 8B | K | DL |
| 41 | 24-Aug | *An. arabiensis* | 200 | 32 | BC | 19:10 | 7:10 | 8A | M | DL |
| 42 | 24-Aug | *An. arabiensis* | 200 | 50 | DM | 19:10 | 7:10 | 8B | K | - |
| 43 | 25-Aug | *An. arabiensis* | 200 | 41 | DM | 19:10 | 7:10 | 8A | M | - |
| 44 | 25-Aug | *An. arabiensis* | 200 | 18 | BC | 19:10 | 7:10 | 8B | K | DL |
| 45 | 26-Aug | *An. arabiensis* | 200 | 33 | BC | 19:05 | 7:00 | 8A | M | DL |
| 46 | 26-Aug | *An. arabiensis* | 200 | 35 | DM | 19:05 | 7:00 | 8B | K | - |
| 47 | 27-Aug | *An. arabiensis* | 200 | 33 | DM | 19:05 | 7:00 | 8A | M | - |
| 48 | 27-Aug | *An. arabiensis* | 200 | 10 | BC | 19:05 | 7:00 | 8B | K | DL |
| 49 | 28-Aug | *An. arabiensis* | 200 | 45 | BC | 19:05 | 7:05 | 8A | M | DL |
| 50 | 28-Aug | *An. arabiensis* | 200 | 34 | DM | 19:05 | 7:05 | 8B | K | - |
| 51 | 29-Aug | *An. arabiensis* | 200 | 38 | DM | 19:00 | 7:05 | 8A | M | - |
| 52 | 29-Aug | *An. arabiensis* | 200 | 32 | BC | 19:00 | 7:05 | 8B | K | DL |
| 53 | 31-Aug | *An. arabiensis* | 200 | 74 | BC | 19:05 | 7:10 | 8A | M | - |
| 54 | 31-Aug | *An. arabiensis* | 200 | 41 | DM | 19:05 | 7:10 | 8B | K | DL |
| 55 | 01-Sep | *An. arabiensis* | 200 | 69 | DM | 19:05 | 7:10 | 8A | M | DL |
| 56 | 01-Sep | *An. arabiensis* | 200 | 32 | BC | 19:05 | 7:10 | 8B | K | - |
| 57 | 02-Sep | *An. arabiensis* | 200 | 69 | BC | 19:05 | 7:05 | 8A | M | - |
| 58 | 02-Sep | *An. arabiensis* | 200 | 63 | DM | 19:05 | 7:05 | 8B | K | DL |
| 59 | 03-Sep | *An. arabiensis* | 200 | 58 | DM | 19:05 | 7:05 | 8A | M | DL |
| 60 | 03-Sep | *An. arabiensis* | 200 | 86 | BC | 19:05 | 7:10 | 8B | K | - |
| 61 | 04-Sep | *An. arabiensis* | 200 | 49 | FL dye | 19:05 | 7:05 | 8A | M | - |
| 62 | 04-Sep | *An. arabiensis* | 200 | 110 | open tubes | 19:05 | 7:05 | 8B | K | DL |
| 63 | 05-Sep | *An. arabiensis* | 200 | 72 | open tubes | 19:05 | 7:05 | 8B | K | DL |
| 64 | 05-Sep | *An. arabiensis* | 200 | 26 | FL dye | 19:05 | 7:05 | 8B | K | - |
| 65 | 06-Sep | *An. arabiensis* | 200 | 54 | BC | 19:00 | 7:05 | 8A | M | - |
| 66 | 06-Sep | *An. arabiensis* | 200 | 58 | DM | 19:00 | 7:05 | 8B | K | DL |
| 67 | 07-Sep | *An. arabiensis* | 200 | 117 | open tubes | 19:10 | 7:05 | 8A | M | DL |
| 68 | 07-Sep | *An. arabiensis* | 200 | 11 | FL dye | 19:10 | 7:05 | 8B | K | - |
| 69 | 08-Sep | *An. arabiensis* | 200 | 60 | FL dye | 21:45 | 7:10 | 8A | P | - |
| 70 | 08-Sep | *An. arabiensis* | 200 | 93 | open tubes | 21:45 | 7:10 | 8B | K | DL |
| 71 | 09-Sep | *An. arabiensis* | 200 | 69 | open tubes | 19:00 | 7:10 | 8A | M | DL |
| 72 | 09-Sep | *An. arabiensis* | 200 | 28 | FL dye | 19:00 | 7:10 | 8B | K | - |
| 73 | 10-Sep | *An. arabiensis* | 200 | 28 | FL dye | 19:02 | 7:00 | 8A | M | - |
| 74 | 10-Sep | *An. arabiensis* | 200 | 75 | open tubes | 19:02 | 7:00 | 8B | K | DL |
| 75 | 11-Sep | *An. arabiensis* | 200 | 90 | open tubes | 19:00 | 7:10 | 8B | K | DL |
| 76 | 11-Sep | *An. arabiensis* | 200 | 35 | FL dye | 19:00 | 7:10 | 8B | M | - |

**Legend**

#Rel = Number of mosquitoes released

#Recap = Number of mosquitoes recaptured the following morning

Time rel = Time released

Time recap = Time recaptured

Screenh = Screen-house number

FL dye = Eave tube treated with fluorescent dye

Open tubes = Eave tube without an eave tube insert

DM = Eave tube insert treated with deltamethrin

BC = Eave tube treated with bendiocarb
